# Gender differences in personality patterns and smoking status after a smoking cessation treatment

**DOI:** 10.1186/1471-2458-13-306

**Published:** 2013-04-08

**Authors:** Bárbara Piñeiro, Ana López-Durán, Elena Fernández del Río, Úrsula Martínez, Elisardo Becoña

**Affiliations:** 1 Smoking Cessation Unit, Department of Clinical Psychology and Psychobiology, Faculty of Psychology, University of Santiago de Compostela, Santiago de Compostela, Spain; 2 Department of Psychology and Sociology, University of Zaragoza, Zaragoza, Spain

## Abstract

**Background:**

The lack of conclusive results and the scarce use of the Millon Clinical Multiaxial Inventory-III (MCMI-III) in the study of the relationship between smoking and personality are the reasons that motivated the study reported here. The aim of the present study was to analyze the influence of personality patterns, assessed with the MCMI-III, and of nicotine dependence on treatment outcomes at the end of the treatment and at 12 months follow-up in men and women smokers receiving cognitive-behavioral treatment for smoking cessation.

**Methods:**

The sample was made up of 288 smokers who received cognitive-behavioral treatment for smoking cessation. Personality patterns were assessed with the Millon Clinical Multiaxial Inventory-III (MCMI-III). Abstinence at the end of the treatment and at 12-month follow-up was validated with the test for carbon monoxide in expired air.

**Results:**

The results showed significant differences by personality patterns that predict nicotine dependence (Narcissistic and Antisocial in men and Schizoid in women). At the end of the treatment it is more likely that quit smoking males with a Compulsive pattern and less likely in those scoring high in Depressive, Antisocial, Sadistic, Negativistic, Masochistic, Schizotypal and Borderline. In women, it is less likely that quit smoking those with the Schizoid pattern. At 12 months follow-up it is more likely that continue abstinent those males with a high score in the Compulsive pattern. Furthermore, nicotine dependence was an important variable for predicting outcome at the end of the treatment and smoking status at 12 months follow-up in both men and women.

**Conclusions:**

We found substantial differences by gender in some personality patterns in a sample of smokers who received cognitive-behavioral treatment for smoking cessation. We should consider the existence of different personality patterns in men and women who seek treatment for smoking cessation.

## Background

In recent years, a large number of publications and studies have focused on the analysis of the relationship between personality traits, from different theoretical models, and cigarette smoking. A part of such research has concentrated on describing which personality traits are most frequently found in smokers [1-3]. For example, Malouff et al. [4], in a meta-analysis of studies carried out from the Big Five-Factor Model, found that smokers score higher on Neuroticism and lower on Agreeableness and Conscientiousness than never-smokers.

Other studies have assessed the extent to which certain personality traits are related to success or failure in smoking cessation and maintaining abstinence [5,6]. For example, Hooten et al. [7] found that according to the Big Five-Factor model, low scores on Neuroticism and Openness were associated with tobacco abstinence. Sher et al. [8] found that Psychoticism and Neuroticism according to Eysenck’s personality model, and Sensation-seeking according to Cloninger’s model, predict relapse. Cosci et al. [9], based on Eysenck’s personality model, report that high scores in Neuroticism and Psychoticism increased risk of relapse in the first year of follow-up after treatment. Furthermore, according to Nieva et al. [10], who made their analysis in the context of the alternative five-factor model [11], smokers’ gender plays a key role in explaining smoking relapse, which in men is related to a high score in Impulsiveness, and in women to a high score in Sociability.

Other research, based on Millon’s personality model, has tried to identify which personality patterns are most common among smokers. Millon defines the personality patterns as a set of attributes that are neither a category nor a dimension, but rather a synthesis of both of them. Patterns that make up the personality of the individual are the results of the inter-relation between contextual and constitutional factors, and are considered to be dynamic styles on a continuum from normality to pathology, which makes possible intervention and change [12]. Normal and abnormal personalities are identified on the basis of Millon’s Evolutionary Personality Theory [13].

Thus, Fernández del Río et al. [14], using the Millon Clinical Multiaxial Inventory-II (MCMI-II), found that smokers scored higher than non-smokers on the Avoidant, Histrionic and Negativistic (Passive-Aggressive) scales. Becoña et al. [15], with the MCMI-III, found the Antisocial and Histrionic patterns to be more frequent in nicotine-dependent smokers, whilst the Schizoid pattern was more common among non-dependent smokers. There are also studies that analyze whether personality patterns influence the results of smoking-cessation treatment. For example, Perea et al. [16] found that smokers with Avoidant, Self-destructive, Passive-Aggressive, Schizotypal or Borderline personality patterns were more likely to relapse at 6 months follow-up, whereas the Dependent pattern was associated with a greater likelihood of maintaining abstinence. In contrast, Fernández del Río et al. [17], using the MCMI-II, found that the Dependent personality pattern was associated with a lower percentage of abstinence at 6-month follow-up.

Therefore, on the basis of Millon’s model, research suggests that certain personality patterns are associated with greater difficulty for smoking cessation and with higher likelihood of relapse, while others would be associated with the maintenance of abstinence. However, the results are still somewhat inconclusive and further research is necessary.

The lack of conclusive results and the scarce use of the MCMI-III [18] in the study of the relationship between smoking and personality are the reasons that motivated the study reported here. Moreover, and as Nieva et al. [10] point out, there are hardly any studies that assess the results of a smoking cessation treatment and the relationship between nicotine dependence, personality and gender. It is necessary to include the variable of nicotine dependence in the studies that assess the results of a treatment to quit smoking, as it is one of the variables that best predicts the results both at the end of the treatment and during the follow-ups [19-21]. Those smokers more nicotine dependent have more difficulties to quit [22,23] and those less dependent are more likely to succeed [24].

The aim of this study is to analyze the influence of the personality patterns, assessed with the MCMI-III, and of nicotine dependence on treatment outcomes at the end of the treatment and at 12 months follow-up in men and women smokers receiving cognitive-behavioral treatment for smoking cessation.

## Methods

### Participants

The sample was made up of smokers seeking smoking cessation treatment at the Smoking Cessation Unit of the Faculty of Psychology at the University of Santiago de Compostela (Spain) during the period January 2009 to June 2011. Participants were recruited through the media, by GPs’ recommendation and by word of mouth from people who had taken the treatment previously. Inclusion criteria were: age 18 or over; a wish to participate in the treatment program; and smoking a minimum of 10 cigarettes a day. Exclusion criteria were: a diagnosis of severe mental disorder (bipolar disorder and/or psychotic disorder); concurrent dependence on other substances (cannabis, cocaine and/or heroin); having participated in the same or similar treatment over the previous year; having received another type of effective smoking cessation treatment (nicotine replacement therapy, bupropion, varenicline) in the past year; suffering from a physical pathology with a high life risk which would require immediate individual intervention (e.g., recent myocardial infarction); smoking a type of tobacco other than cigarettes (e.g., cigars); refusing to be video-recorded during the sessions; and failing to attend the first treatment session.

From an initial sample of 332 smokers, 42 were excluded for having fulfilled one of the exclusion criteria and two because their MCMI-III score was invalid (validity scale score = 3). Therefore the final sample was made up of 288 smokers (41% men, 59% women). Their average age was 41.49 years (*SD* = 10.55).

### Measures

All the smokers were assessed with the Smoking Habit Questionnaire (*Cuestionario sobre el hábito de fumar*[25]), which collects information on sociodemographic variables (gender, age) and about smoking (number of cigarettes smoked pre-treatment).

For the assessment of personality patterns we used the Millon Clinical Multiaxial Inventory-III (MCMI-III [18]; Spanish adaptation by Cardenal et al. [26]). This is a 175-item self-report scale with two response options (true-false) that assesses 14 personality patterns and 10 clinical syndromes. The personality patterns assessed are: Schizoid, Avoidant, Depressive, Dependent, Histrionic, Narcissistic, Antisocial, Sadistic (Aggressive), Compulsive, Negativistic (Passive-Aggressive), Masochistic (Self-Defeating), Schizotypal, Borderline, and Paranoid. On the MCMI-III, raw scores are transformed into prevalence scores (PREV), and such scores are used in two ways: 1) a PREV score of 75 and 85 is considered to indicate clinical personality traits, while scores of 85 and over indicate a chronic and moderately severe level of functioning, a personality disorder [27,28]; and 2) PREV are used as continuous scores without applying cut-off points [29,30]. In the original sample, the Cronbach’s alpha coefficients of the clinical scales range between 0.66 and 0.90; in the standardized Spanish scale (N = 964) they range from 0.65 to 0.88, being, in general, very similar to those obtained in US population [26].

For the assessment of nicotine dependence we used the Fagerström Test for Nicotine Dependence (FTND [31]), in its validated Spanish version by Becoña et al. [32]. This scale is made up of 6 items and it is the most widely used instrument for rating nicotine dependence. In the present sample the reliability obtained by means of Cronbach’s alpha was 0.65.

We used the Micro^+^ Smokerlyzer® (Bedfont Scientific Ltd, Sittingbourne, England) to measure carbon monoxide (CO) in expired air, so as to corroborate self-reported abstinence at the end of the treatment and at 12-month follow-up.

### Procedure

Initial assessment of the smokers was carried out in a single session in which all the smokers gave their informed consent for participation in the study. The study was authorized by the Bioethics Committee of the University of Santiago de Compostela.

The cognitive-behavioral treatment applied was the Smoking Cessation Program (*Programa para Dejar de Fumar,*[33]). This is a cognitive-behavioral treatment has demonstrated its efficacy in previous studies [34]. It is composed by six sessions (one per week of about 1 hour), applied in group format. This treatment program involves components of treatment contract, self-monitoring of smoking, and graphic representation of cigarette consumption, general information about tobacco, nicotine fading, stimulus control, activities for avoiding nicotine-withdrawal syndrome, physiological feedback on cigarette consumption, and relapse-prevention strategies (assertiveness, problem solving, changes in erroneous beliefs, anxiety and anger management, physical exercise, weight control, and self-support). The groups were composed by 6–8 participants and the assignment to them was done based on their schedule availability. Sociodemographic or consumption characteristics were not taken into account to this end. Groups were conducted by four different psychologists and no differences were found on treatment results according to the therapist of the group.

After the treatment there was face-to-face follow-up at 12 months. Self-report of abstinence at the end of the treatment and in the follow-up was tested by measures of carbon monoxide (CO) in expired air, using the Micro^+^ Smokerlyzer®. The West et al. [35] criteria were adopted for point prevalence abstinence at the end of the treatment (not having smoked in the past 24 hours, CO < 10 ppm) and continuous abstinence at 12 months follow-up (not having smoked, not even a puff, since the end of the treatment, CO < 10 ppm).

In the follow-up, in those cases in which it was not possible to locate the participants, they were considered to be smokers, and at the same level (in terms of number of cigarettes and nicotine content) as in the pretreatment assessment.

### Data analysis

First, a descriptive analysis was made of the total sample (N = 288). Next, we analyzed the correlations between personality patterns and measures of nicotine dependence (FTND score and number of cigarettes smoked per day). In order to test differences between abstainers and smokers at the end of the treatment and at the 12-month follow-up or between gender, we used Student’s *t*-tests and Cohen’s d to estimate the effect size. We also examined the contribution of personality patterns to nicotine dependence (according to FTND score) using a stepwise multiple regression analysis. In the next part of the analysis we examined the contribution of personality patterns to treatment outcomes. For this step, a stepwise logistic regression analysis was carried out to examine personality patterns as predictors of smoking cessation at the end of the treatment and at the 12-month follow-up, adjusted for initial levels of nicotine dependence. All statistical analyses were performed using the SPSS software (19.0; © SPSS Inc., 2010). The significance level was set at p < 0.05.

## Results

Participants (N = 288; 41% men, 59% women) smoked a mean of 21.7 cigarettes/day (*SD* = 8.37, range 10–50), obtaining a mean FTND score of 5.07 (*SD* = 2.19, range 0–10). As far as smoking rate is concerned, women smoked fewer cigarettes/day than men while no significant gender differences were found regarding the level of nicotine dependence (Table 1).

**Table 1:** Descriptive statistics in smoking, nicotine dependence and personality pattern variables

	**Total (N = 288)**		**Men (N = 118)**		**Women (N = 170)**		
	**Mean**	**SD**	**Mean**	**SD**	**Mean**	**SD**	***t***
**CPD**	21.70	8.37	23.07	8.74	20.75	7.99	2.33*
**FTND**	5.07	2.19	4.94	2.34	5.16	2.07	−0.83
Personality patterns							
**Schizoid**	38.75	20.47	37.63	20.63	39.53	20.39	−0.76
**Avoidant**	35.95	24.55	35.87	24.07	36.01	24.95	−0.05
**Depressive**	31.88	25.72	30.22	24.97	33.03	26.23	−0.91
**Dependent**	37.45	23.81	37.89	21.56	37.15	25.31	0.26
**Histrionic**	55.77	21.09	50.14	19.62	59.68	21.24	−3.87***
**Narcissistic**	60.01	16.43	67.27	13.00	54.98	16.71	6.71***
**Antisocial**	46.41	20.25	43.88	20.66	48.17	19.84	−1.77
**Sadistic (Aggressive)**	42.93	21.30	42.32	22.71	43.35	20.32	−0.40
**Compulsive**	58.08	18.42	54.20	18.18	60.76	18.15	−3.02**
**Negativistic (Passive-Aggressive)**	39.92	22.18	40.28	22.42	39.66	22.08	0.23
**Masochistic (Self-Defeating)**	28.28	23.17	25.94	22.64	29.91	23.47	−1.43
**Schizotypal**	24.03	24.43	23.88	24.38	24.14	24.54	−0.09
**Borderline**	34.03	22.64	33.12	22.93	34.66	22.48	−0.57
**Paranoid**	34.73	25.53	34.85	24.63	34.65	26.21	0.07

CPD = Cigarettes per day; FTND = Fagerström Test for Nicotine Dependence.

*p < .05; **p < .01; ***p < .001.

When analyzing the scores on MCMI-III personality patterns, we found that women scored significantly higher than men on the Histrionic and Compulsive patterns, whilst men scored higher on the Narcissistic pattern (see Table 1).

Of the 288 smokers in the study, 58% (n = 167) had stopped smoking by the end of the treatment and this proportion of abstainers were confirmed through the carbon monoxide in expired air measure (CO < 10 ppm). At 12 months follow-up, 19.8% (n = 57) of the sample were abstinent.

### Relationship between personality patterns and nicotine dependence

Table 2 shows the bivariate correlations between the 14 personality patterns of the MCMI-III, scores on the FTND and pre-treatment cigarettes/day. In the relationship between nicotine dependence and MCMI-III personality patterns in the total sample we found a positive relationship between nicotine dependence and the Schizoid, Depressive, Sadistic (Aggressive), Negativistic, Schizotypal, and Borderline patterns; and a negative relationship with the Histrionic pattern. This relationship varies according to smoker’s gender. In the case of women, we found a positive relationship between nicotine dependence and the Schizoid, Sadistic (Aggressive), Negativistic, and Paranoid patterns; and a negative relation with the Histrionic pattern. In the case of men, we only found a positive correlation between FTND score and the Schizoid and Antisocial patterns, together with a negative correlation with the Narcissistic pattern. Nevertheless, we have to note that some correlations between nicotine dependence (score on FTND) and personality patterns were quite low (between -.12 and .18), even though statistically significant. It is consistent with the low percentage of variance explained by the regression model.

**Table 2:** Correlations between personality patterns, nicotine dependence (FTND) and number of cigarettes smoked per day (CPD)

	**1**	**2**	**3**	**4**	**5**	**6**	**7**	**8**	**9**	**10**	**11**	**12**	**13**	**14**	**FTND**	**CPD**
**1. Schizoid**	-	.58**	.49**	.26**	-.69**	-.46**	.11	.22**	-.10	.39**	.45**	.52**	.38**	.41**	.18**	.04
**2. Avoidant**	.61** (.57**)	-	.67**	.58**	-.70**	-.65**	.13*	.22**	-.14*	.49**	.62**	.63**	.50**	.47**	.10	-.04
**3. Depressive**	.39** (.55**)	.56** (.75**)	-	.62**	-.50**	-.54**	.24**	.34**	-.26**	.59**	.73**	.67**	.68**	.51**	.13*	.02
**4. Dependent**	.24** (.27**)	.54** (.60**)	.55** (.66**)	-	-.33**	-.45**	.18**	.26**	-.17**	.54**	.65**	.53**	.53**	.44**	.05	.04
**5. Histrionic**	-.73** (−.72**)	-.72** (−.72**)	-.38** (−.62**)	-.27** (−.37**)	-	.53**	-.05	-.21**	.17**	-.38**	-.41**	-.56**	-.37**	-.40**	-.12*	-.04
**6. Narcissistic**	-.47** (−.49**)	-.62** (−.75**)	-.46** (−.62**)	-.45** (−.51**)	.57** (.73**)	-	-.11	-.11	.04	-.37**	-.48**	-.41**	-.38**	-.27**	-.11	.04
**7. Antisocial**	.25** (.01)	.12 (.13)	.23** (.23**)	.14 (.20**)	-.14 (−.04)	-.02 (−.11)	-	.69**	-.61**	.52**	.32**	.27**	.55**	.26**	.10	.01
**8. Sadistic-Aggressive**	.30** (.16*)	.25** (.20**)	.34** (.35**)	.25** (.27**)	-.29** (−.17*)	-.09 (−.13)	.75** (.64**)	-	-.47**	.66**	.43**	.40**	.61**	.45**	.13*	.06
**9. Compulsive**	-.18* (−.06)	-.09 (−.18*)	-.25** (−.28**)	-.11 (−.20**)	.15 (.12)	-.01 (.18*)	-.63** (−.65**)	-.46** (−.50**)	-	-.44**	-.28**	-.29**	-.49**	-.18**	-.04	-.02
**10. Negativistic**	.41** (.37**)	.44** (.52**)	.50** (.65**)	.54** (.54**)	-.32** (−.44**)	-.36** (−.43**)	.53** (.53**)	.66** (.67**)	-.38** (−.49**)	-	.64**	.57**	.79**	.65**	.13*	.04
**11. Masochistic**	.44** (.45**)	.51** (.70**)	.66** (.77**)	.59** (.69**)	-.37** (−.48**)	-.39** (−.54**)	.43** (.23**)	.52** (.36**)	-.33** (−.28**)	.67** (.63**)	-	.63**	.69**	.50**	.08	.01
**12. Schizotypal**	.49** (.54**)	.61** (.64**)	.62** (.70**)	.56** (.52**)	-.52** (−.60**)	-.38** (−.47**)	.37** (.20**)	.52** (.30**)	-.32** (−.28**)	.59** (.57**)	.62** (.64**)	-	.63**	.54**	.13*	.09
**13. Borderline**	.38** (.37**)	.42** (.56**)	.62** (.72**)	.49** (.56**)	-.31** (−.44**)	-.34** (−.43**)	.54** (.55**)	.63** (.60**)	-.45** (−.55**)	.75** (.82**)	.69** (.69**)	.67** (.60**)	-	.50**	.14*	.07
**14. Paranoid**	.48** (.36**)	.53** (.43**)	.46** (.53**)	.47** (.42**)	-.37** (−.43**)	-.32** (−.27**)	.33** (.22**)	.46** (.45**)	-.16 (−.19*)	.61** (.69**)	.49** (.51**)	.53** (.54**)	.47** (.52**)	-	.11	-.01
**FTND**	.19* (.17*)	.07 (.11)	.14 (.12)	.01 (.09)	-.10 (−.16*)	-.20* (−.05)	.19* (.02)	.10 (.16*)	-.15 (.03)	.10 (.16*)	.04 (.10)	.11 (.14)	.14 (.14)	.05 (.16*)	-	.61**
**CPD**	.02 (.07)	-.03 (−.04)	.06 (.01)	-.01 (.06)	.02 (−.03)	-.07 (.02)	-.10 (−.03)	-.05 (.08)	-.09 (.08)	.04 (.05)	.05 (−.01)	.09 (.09)	.07 (.08)	-.07 (.04)	.69** (.56**)	-

FTND = Fagerström Test for Nicotine Dependence; CPD = Cigarettes per day;

The upper right quadrant shows the correlations obtained with the total sample (N = 288). The lower left quadrant shows the correlations according to gender (correlations in the women’s group in brackets).

*p < .05; **p < .01.

We found no relationship between number of cigarettes/day and all the personality patterns. As the results were different according to gender, we decided to perform further analyses for men and women separately.

In a second step, stepwise multiple regression was performed by gender, including the 14 personality patterns. Two personality patterns, Narcissistic (B = −0.04) and Antisocial (B = 0.02), were involved in the model (F = 4.59, p < .05), explaining 5.8% of FTND variance for men. For women, only the Schizoid personality pattern (B = 0.02) was involved in the model (F = 4.68, p < .05), explaining 2.1% of the variance.

### Relationship between personality patterns and treatment outcomes in men and women

Men who did not manage to give up smoking by the end of the treatment scored higher in the Depressive, Antisocial, Sadistic (Aggressive), Negativistic, Masochistic, Schizotypal and Borderline patterns, and those who did manage to give up smoking scored higher in the Compulsive pattern. In the women’s group we only found differences in the Schizoid pattern, in which those who did not give up smoking by the end of the treatment scored higher (Table 3).

**Table 3:** Mean scores (standard deviations) in the personality patterns and results at the end of the treatment

	**Men (N = 118)**				**Women (N = 170)**				**Total (N = 288)**			
**Personality patterns**	**Abstinent (n = 63)**	**Smokers (n = 55)**	**t**	**Cohen’s d**	**Abstinent (n = 104)**	**Smokers (n = 66)**	**t**	**Cohen’s d**	**Abstinent (n = 167)**	**Smokers (n = 121)**	**t**	**Cohen’s d**
**Schizoid**	36.19 (18.83)	39.27 (22.58)	−0.81		36.85 (20.20)	43.76 (20.11)	−2.18*	0.17	36.60 (19.64)	41.72 (21.29)	−2.11*	0.12
**Avoidant**	33.44 (23.16)	38.65 (25.01)	−1.18		34.41 (23.39)	38.52 (27.22)	−1.05		34.05 (23.23)	35.58 (26.13)	−1.55	
**Depressive**	23.59 (21.99)	37.82 (26.17)	−3.21**	0.30	32.16 (25.08)	34.39 (28.10)	−0.54		28.93 (24.26)	35.95 (27.18)	−2.31*	0.15
**Dependent**	35.02 (20.55)	41.18 (22.39)	−1.56		36.65 (25.97)	37.94 (24.42)	−0.32		36.04 (24.01)	39.41 (23.48)	−1.19	
**Histrionic**	51.46 (18.29)	48.64 (21.11)	0.78		61.24 (20.20)	57.23 (22.72)	1.20		57.55 (20.02)	53.32 (22.33)	1.69	
**Narcissistic**	68.67 (11.40)	65.67 (14.57)	1.25		56.87 (15.43)	52.00 (18.27)	1.86		61.32 (15.14)	58.21 (17.97)	1.59	
**Antisocial**	39.29 (20.83)	49.15 (19.33)	−2.65**	0.24	48.16 (19.21)	48.18 (20.94)	−0.01		44.81 (20.24)	48.62 (20.14)	−1.58	
**Sadistic (Aggressive)**	37.71 (23.97)	47.60 (20.11)	−2.41*	0.22	43.36 (20.83)	43.35(19.65)	0.01		41.23 (22.16)	45.28 (19.89)	−1.60	
**Compulsive**	58.54 (17.54)	49.24 (17.77)	2.86**	0.26	62.64 (16.16)	57.80 (20.69)	1.71		61.10 (16.76)	53.91 (19.81)	3.33**	0.19
**Negativistic (Passive-Aggressive)**	36.35 (22.07)	44.78 (22.15)	−2.07*	0.19	39.41 (22.48)	40.06 (21.60)	−0.19		38.26 (22.31)	42.21 (21.89)	−1.49	
**Masochistic (Self-Defeating)**	20.05 (20.52)	32.69 (23.25)	−3.14**	0.29	29.04 (23.54)	31.29 (23.46)	−0.61		25.65 (22.81)	31.93 (23.27)	−2.29*	0.13
**Schizotypal**	18.24 (21.71)	30.35 (25.83)	−2.77**	0.26	22.88 (24.38)	26.12 (24.83)	−0.84		21.13 (23.45)	28.04 (25.27)	−2.39*	0.14
**Borderline**	27.44 (21.88)	39.62 (22.56)	−2.97**	0.27	33.12 (21.97)	37.09 (23.23)	−1.12		30.98 (22.04)	38.24 (22.87)	−2.72**	0.16
**Paranoid**	33.37 (23.56)	36.55 (25.93)	−0.70		35.32 (25.76)	33.59 (27.07)	0.42		34.58 (24.90)	34.93(26.49)	−0.12	

*p < .05; **p < .01.

Regarding smoking status at 12 months follow-up we only found significant differences in the men’s group: those who were abstinent scored higher in the Compulsive pattern [Mean = 61.23 (*SD* = 18.66) for abstainers and 52.59 (*SD* = 17.78) for smokers; t = 2.04, df = 168, p < .05].

### Personality patterns as predictors of smoking cessation and maintenance of abstinence in men and women

We carried out a stepwise logistic regression, taking as predictor variables the scores on the MCMI-III personality patterns, adjusted for levels of nicotine dependence (based on initial FTND scores) separated according to gender. The criterion variable was “abstinence” at the end of the treatment and at the 12-month follow-up (1 = Yes, 0 = No).

At the end of the treatment, in women none of the 14 personality patterns predicted abstinence, and only nicotine dependence (FTND) appears as a predictor variable. In men, the Masochistic personality pattern and FTND were associated with abstinence at the end of the treatment. Men and women with higher nicotine dependence were less likely to give up smoking. In the case of men, we also found that those who score higher in the Masochistic pattern were less likely to achieve abstinence (Table 4).

**Table 4:** Logistic regression analysis at the end of the treatment and at 12-month follow-up, by gender

**Time**	**B**^**a**^	**Wald**	**p**	**OR**	**C.I. (95%)**
**Men (n = 118)**					
Criterion variable = Abstinence at the end of treatment					
Masochistic (Self-Defeating)	-.028	8.962	.003	.972	.955-.990
FTND	-.319	11.334	.001	.727	.604-.875
Criterion variable = Abstinence at 12-month follow-up					
FTND	−0.276	5.875	.015	.759	.607-.949
**Women (n = 170)**					
Criterion variable = Abstinence at the end of treatment					
FTND	-.236	8.195	.004	.789	.671-.928
Criterion variable = Abstinence at 12-month follow-up					
FTND	-.182	3.820	.051	.833	.694-1.000

^a^ Coding of the groups in the model was Abstinent = 1 and Smokers = 0.

In continuous abstinence at 12 months follow-up, in both women and men, no personality pattern was involved in the model, and the only significant predictor of continuous abstinence was nicotine dependence (Table 4).

## Discussion

The aim of this study was to analyze the influence of personality patterns assessed with the MCMI-III and nicotine dependence on smoking status (at the end of the treatment and at 12 months follow-up) in a sample of 288 men and women smokers receiving cognitive-behavioral treatment for smoking cessation. The results indicated differences between men and women in the personality patterns analyzed in the present study, and that nicotine dependence plays a relevant role in treatment outcomes.

When assessing the personality patterns of smokers seeking smoking cessation treatment, we found that women score significantly higher in the Histrionic and Compulsive patterns, whilst in men the Narcissistic pattern is the most prevalent. This result is in line with those of other studies, such as that of Berlin et al. [36], who concluded that there are different reasons for smoking in men and in women: women smoked more for tension reduction/relaxation, stimulation and social reasons than men. In a similar line, Millon [37] argues that the Compulsive pattern is characterized by continuous tension, and the Histrionic pattern by a need for continual social reinforcement.

We did find differences regarding to which patterns predict such dependence. In men, nicotine dependence was predicted by a high score in the Antisocial pattern and a low score in the Narcissistic pattern, and in women it was predicted by a high score in the Schizoid personality pattern. Nieva et al. [10] had already found that low Sociability predicts nicotine dependence in men, but in the women’s group no personality scale emerged as a significant predictor of nicotine dependence.

On the other hand, we did find differences by gender in relation to the patterns most strongly related to smoking abstinence at the end of the treatment. Men with a high score in the Compulsive pattern were likely to stop smoking, but high scores in the Depressive, Antisocial, Sadistic, Negativistic, Masochistic, Schizotypal and Borderline personality patterns were associated with lower percentages of abstinence at the end of the treatment. The Antisocial and Borderline patterns have in common the Impulsiveness trait, which according to Nieva et al. [10] is related to smoking relapse in men. The only personality pattern associated with treatment outcomes in women was Schizoid. Women with a high score on this personality pattern were less likely to stop smoking. According to the results obtained in the present study, this pattern is related to a higher nicotine dependence in women who seek treatment and nicotine dependence is related to higher difficulties to quit smoking [19].

Long-term outcomes were also different according to personality patterns. In men, a high score in the Compulsive pattern was related to continuous abstinence at 12-months follow-up, while in women no pattern was significant. Previous studies carried out from Millon’s personality model [12], without taking into account gender, also found good results among smokers with this personality pattern. A plausible explanation is that people with this pattern are highly perfectionist and inflexible in their decisions, an example of which would be the decision to attend a specific treatment smoking cessation program. If we bear in mind that the criterion in the present study for classifying a participant as abstinent at 12-months follow-up was not having smoked a cigarette, not even a puff, since the end of the treatment, it would not be surprising to find that participants with this personality pattern are those with the highest probability of remaining abstinent in the long term. In the same line, another reflection of the high level of control in people with this pattern are the results of studies carried out with psychoactive substance users, in which this personality pattern shows low prevalence [38,39].

Concerning the regression analysis, nicotine dependence is the determining variable for smoking status in both short and long term, regardless of gender. In men only, the Masochistic pattern partly explains poor outcomes at the end of the treatment. Nicotine dependence is considered the key variable for explaining the results of smoking cessation interventions according to previous studies [19,40,41], which have concluded that people with higher nicotine dependence have more problems to maintain long term abstinence.

However, the results also indicated that certain personality patterns predict nicotine dependence and that there were significant differences according to gender. On the one hand, the Schizoid pattern has been linked to smoking at the end of the treatment in women, and on the other hand, higher scores in this pattern was associated with higher nicotine dependence. The Antisocial personality pattern has been linked to lower percentages of abstinence, and has been a pattern that contributes to explain the variance of FTND in men. Thus, we know that certain personality patterns, which differ according to gender (e.g., Schizoid in women), are related to greater dependence, which in turn predicts poorer results both at the end of the treatment and at the 12-month follow-up.

Some limitations in this study should be noted. First, the sample is not representative of the total population of smokers, since it includes only those who sought smoking cessation treatment, and we know that this type of population has different characteristics from those of smokers from the general population. Therefore, we could expect that the patterns and the differences found by gender could vary in the profile of smokers who stop smoking without following a specific treatment, so that our results could be extrapolated only to similar clinical samples. As Hughes et al. [42] and Le Strat et al. [43] point out, the results obtained in studies with smokers who seek treatment are not generalizable to smokers of the general population due to the particular characteristics of persons who seek treatment. Thus, it would be necessary to conduct studies out of clinical samples to know how results would be. Secondly, the instrument used for the assessment of personality patterns, the MCMI-III, is based on a specific theoretical perspective. Theodore Millon’s personality model has large numbers of both advocates and detractors, but despite its critics it is one of the most widely used with clinical population in the study of personality [44,45]. However, to our knowledge, this is the first study which examines the relationship between personality according to Millon’s model and smoking cessation outcomes in a large clinical sample, taking into account the contribution of gender to this relationship.

## Conclusions

In summary, as far as we know, this is the first study which examines the relationship between Millon’s personality patterns and smoking status in a clinical sample taking into account the influence of gender. Our results show that the personality patterns of men and women who seek treatment to quit smoking are different, and also that there are significant differences between personality patterns and nicotine dependence and their influence on treatment outcomes by gender. However, only nicotine dependence had a significant role in predicting continuous abstinence at the 12-month follow-up. But as Marqueta et al. [46] indicate, although the gender variable may not explain much in the models predicting the results of smoking cessation interventions, it should be taken into account for two reasons. First, because gender is related to a determinant factor of treatment success, which is nicotine dependence. And second, because the personality profiles of smokers who seek treatment for smoking cessation are different in men and in women. Berlin et al. [36] even propose designing specific interventions for men and women, so that we would have to take into account their characteristics if we are to improve smoking cessation outcomes.

## Competing interests

The authors declare that they have no competing interests.

## Author’s contributions

BP, EB designed the study and wrote the protocol. BP, ALD, EFR, UM conducted literature searches and provided summaries of previous research studies. BP, ALD, EFR, UM conducted the statistical analysis. BP, ALD, EFR wrote the first draft of the manuscript, and all authors contributed to and have approved the final manuscript.

## Pre-publication history

The pre-publication history for this paper can be accessed here:

http://www.biomedcentral.com/1471-2458/13/306/prepub
